# Diagnostic Algorithm for Glycogenoses and Myoadenylate Deaminase Deficiency Based on Exercise Testing Parameters: A Prospective Study

**DOI:** 10.1371/journal.pone.0132972

**Published:** 2015-07-24

**Authors:** Fabrice Rannou, Arnaud Uguen, Virginie Scotet, Cédric Le Maréchal, Odile Rigal, Pascale Marcorelles, Eric Gobin, Jean-Luc Carré, Fabien Zagnoli, Marie-Agnès Giroux-Metges

**Affiliations:** 1 Physiology Department-EA 1274, CHRU Cavale Blanche, Brest, France; 2 Pathology Department, CHRU Morvan, Brest, France; 3 Institut National de la Santé et de la Recherche Médicale, UMR 1078, Brest, France; 4 Biochemistry Department, Robert Debré Hospital-APHP, Paris, France; 5 Pathology Department-EA 4685 LNB, CHRU Morvan, Brest, France; 6 Biochemistry Department, CHRU Cavale Blanche, Brest, France; 7 Neurology Department-EA 4685 LNB, Clermont-Tonnerre Armed Forces Hospital, Brest, France; VU University Medical Center, NETHERLANDS

## Abstract

**Aim:**

Our aim was to evaluate the accuracy of aerobic exercise testing to diagnose metabolic myopathies.

**Methods:**

From December 2008 to September 2012, all the consecutive patients that underwent both metabolic exercise testing and a muscle biopsy were prospectively enrolled. Subjects performed an incremental and maximal exercise testing on a cycle ergometer. Lactate, pyruvate, and ammonia concentrations were determined from venous blood samples drawn at rest, during exercise (50% predicted maximal power, peak exercise), and recovery (2, 5, 10, and 15 min). Biopsies from vastus lateralis or deltoid muscles were analysed using standard techniques (reference test). Myoadenylate deaminase (MAD) activity was determined using *p*-nitro blue tetrazolium staining in muscle cryostat sections. Glycogen storage was assessed using periodic acid-Schiff staining. The diagnostic accuracy of plasma metabolite levels to identify absent and decreased MAD activity was assessed using Receiver Operating Characteristic (ROC) curve analysis.

**Results:**

The study involved 51 patients. Omitting patients with glycogenoses (n = 3), MAD staining was absent in 5, decreased in 6, and normal in 37 subjects. Lactate/pyruvate at the 10th minute of recovery provided the greatest area under the ROC curves (AUC, 0.893 ± 0.067) to differentiate Abnormal from Normal MAD activity. The lactate/rest ratio at the 10th minute of recovery from exercise displayed the best AUC (1.0) for discriminating between Decreased and Absent MAD activities. The resulting decision tree achieved a diagnostic accuracy of 86.3%.

**Conclusion:**

The present algorithm provides a non-invasive test to accurately predict absent and decreased MAD activity, facilitating the selection of patients for muscle biopsy and target appropriate histochemical analysis.

## Introduction

Myalgia and exercise intolerance are common complaints in clinical practice. The symptoms may represent inflammatory myopathies, muscular dystrophies, congenital myopathies and metabolic myopathies, as well as non-myopathic conditions. Following clinical examination, the diagnostic method is challenging, and investigation screening mostly involves electromyogram, serum creatine kinase, and magnetic resonance imaging [1–3]. A definitive diagnosis requires an invasive muscle biopsy and highly specialised techniques for analysis. However, an initial non invasive test could be useful to promote rigorous selection of patients for muscle biopsy and to select the appropriate analysis [1–4].

Exertional symptoms are the hallmarks of metabolic myopathies, supporting the concept of using functional tests when this diagnosis is suspected [5–9]. Exercise increases the concentrations of muscle metabolites in the venous blood supply (e.g. lactate, pyruvate, and ammonia), especially during recovery [7,10]. Thus, venous blood sampling during and after exercise is an easy method to provide information about muscle metabolism. In mitochondrial myopathies, the determination of lactate concentrations at rest or following exercise has been proposed as a diagnosis tool [6–8,11]. Myoadenylate deaminase (MAD) deficiency is by far the most frequent metabolic myopathy [6,12–17]. MAD catalyses the irreversible hydrolytic conversion of AMP into IMP with the concurrent release of ammonia. During forearm ischemic testing the failure to produce ammonia with increase in plasma lactate levels is a characteristic feature of MAD deficiency (MADD)[5,14–16,18–20]. Cardiopulmonary exercise testing (CPX) provides an objective measurement of peak functional capacity and assesses the pulmonary, cardio-vascular, and skeletal muscle adaptation to exercise. Incremental exercise testing is used in most institutions as a diagnostic tool for cardiac and lung diseases. In contrast, few studies have evaluated the diagnostic accuracy of incremental exercise testing to investigate metabolic myopathies [6,11]. To assess the validity of this approach, we therefore evaluated metabolic parameters of blood sampled during and after CPX against the reference standard, i.e. muscle biopsy, in order to develop an intuitive non-invasive diagnostic algorithm for metabolic myopathies. To fine-tune the accuracy of our decision tree, we used Receiver Operating Characteristic (ROC) curve analysis to validate each node of the decision tree and to determine cut-off values [21–26].

## Materials and Methods

### Study population

This study was approved by the Brest University Hospital Ethics Committee and was performed in compliance with the standards set by the latest revision of the declaration of Helsinki. Before inclusion, written informed consent was obtained from all patients and, if they were minors, from their legal representatives. This observational study was performed at Brest Hospital according to the Standards for Reporting of Diagnostic Accuracy (STARD) recommendation. All the consecutive patients (aged > 16 years) attending a metabolic exercise testing were prospectively enrolled from December 2008 to September 2012 (Physiology Department, Brest Hospital). During this period, the patients that also performed the reference test, that is muscle biopsy, were also included. Our goal was to diagnose the following four types of metabolic myopathies [27,28]: Mitochondrial respiratory chain deficiencies, lipid oxidation disorders, glycogenoses, and myoadenylate deaminase deficiency.

### Exercise protocol

Patients were instructed to avoid exercise and alcohol consumption for 48 hours before exercise testing. The patients reported to the laboratory after an overnight fast (12−14 h) in order to control dietary status, resting metabolite concentrations [5,19], and determine plasma carnitine and acylcarnitine profile. A urine sample was collected to perform organic acids analysis.

Subjects performed an incremental exercise test using a cycle ergometer (Ergoline GmbH, Bitz, Germany) operated via a MedGraphic CPX (Medical Graphics Corporation, St. Paul, MN). Before each test, the inspiratory flow meter was calibrated with a 3−L volume syringe (Hans Rudolph Inc., Kansas City, MO) and the V’O_2_ and V’CO_2_ gas analysers were calibrated using high-precision gases (16.00 ± 0.04% O_2_ and 5.00 ± 0.10% CO_2_, Air Liquide Healthcare, Plumsteadville, PA). Heart rate (HR) was measured from the electrocardiogram and expressed as the percentage of the predicted value (220—age). Predicted maximal power (PMP) was calculated using anthropometric data as previously described [29] with adjustment according to the exercise intolerance [30]. In order to exhaust the subject in 10–12 min, the initial 2-min workload and subsequent 1-min increments were set at 20 and 10% of PMP, respectively. Maximal effort was defined as the inability to sustain the required pedalling frequency (60 revolutions/min) in spite of vigorous verbal encouragement. Peak V’O_2_ was defined as the mean of the highest two consecutive values of 15-second averages of V’O_2_. To further compare exercise testing parameters between subjects, their measured peak V’O_2_ and maximal power were expressed as percentages of the predicted values (Wasserman [10] and Jones [29], respectively).

### Blood samples

Before exercise, an 18-gauge catheter was placed in a left antecubital vein [6,7,9,31,32] to allow peripheral access for repeated blood draws (n = 7). A continuous flow of normal saline (15 ml/h) was provided to the catheter. Venous blood was first sampled at rest in supine position. During exercise, blood was collected twice, when the V’CO_2_/V’O_2_ ratio (Respiratory Exchange Ratio, RER) reached 1 or, at the latest, at 50% PMP, and at peak exercise. Blood was withdrawn 2, 5 10, and 15 min after exercise.

For lactate and pyruvate measurement, 1 mL blood was withdrawn in a tube containing 2 mL perchloric acid 1M previously cooled to 0°C. For ammonia determination, 4 mL venous blood was collected into a heparinized tube. All collected samples were promptly cooled on dry ice. Blood specimens for the determination of lactate and pyruvate levels were stored at -80°C until analysis.

### Blood metabolite analysis

Ammonia was determined immediately following sampling using an enzymatic and spectrophotometric assay [33] on an automated clinical analyzer (ADVIA 1800, Siemens Healthcare Diagnostics Inc., NY). Lactate concentration was assayed spectrophotometrically using the lactate oxidase method. Pyruvate was measured by enzymatic assay on the basis of the intrinsic extinction coefficient of NADH at 340 nM in the presence of lactate dehydrogenase.

### Biopsy Histoenzymology

Muscle biopsies were evaluated in a reference laboratory (Pathology Department, Brest Hospital), processing about two hundreds biopsies/year. Samples were obtained from vastus lateralis or deltoid muscles using the open biopsy method, and subsequent analysis of serial cryostat sections involved standard techniques: hematoxylin and eosin (HE), adenosinetriphosphatase (ATPase) with preincubation at pH 9.4, 4.63, and 4.3, modified Gomori trichrome, periodic acid-Schiff (PAS), phosphorylase, Sudan black, cytochrome c oxidase-succinate dehydrogenase (COX-SDH), and MAD staining.

MAD activity was determined in 10−μm thick transverse muscle sections using *p*-nitro blue tetrazolium stain according to the method developed by Fishbein et al. [34,35]. Slides were incubated for 1 h at room temperature (22 ± 2°C) in a medium containing 0.2 M potassium chloride (KCl), 1.2 M adenosine monophosphate (AMP), 3.2 mM *p-*nitro-blue tetrazolium (*p*-NBT), and 0.1 M dithiothreitol at pH 6.1. The sections were then washed with distilled water and embedded in glycerol jelly.

In order to obtain a quantitative assessment of histoenzymatic staining [36], the optical density (OD) of *p*-NBT staining was measured. Stained muscle sections were examined with a microscope and digital images were captured using a colour camera (Olympus BX51, Hamburg, Germany). For each muscle section, light transmittance of the stained slices (I) was measured (Mesurim Pro Software, jean-francois.madre@ac-amiens.fr), and OD (given as %) was calculated.

Biopsy specimens from at least three patients were processed together under strictly similar conditions using the same reagents. The OD in studied subjects was expressed as a percentage of mean OD in the other muscle biopsies processed at the same time. On this basis, MAD staining was classified as Absent, Decreased or Normal [13,15,16,18,34,37–39]. Criteria for decreased histochemical MAD activity was abnormal low staining intensity and undistinguishable fibre types [40]. Absent and decreased MAD stainings were checked in another series of analysis. OD analysis (reference test) was performed by trained scientists who were unaware of exercise testing results (index test).

### Statistics

Quantitative variable values are expressed as means ± standard deviation (SD) unless otherwise indicated. A one-way ANOVA was conducted to evaluate differences between groups for MAD staining (OD), CPX parameters, and blood metabolite data. When significance was indicated (*P* < 0.05), the Games-Howell *post-hoc* procedure was applied to identify differences between groups. Statistical analysis was undertaken using SAS software (ver. 9.2, SAS Institute Inc, Cary, NC). The criterion of statistical significance was set at *P* < 0.05.

### Algorithm development

ROC curves [21–23] were generated to determine a decision tree algorithm for MAD deficiency following a step-by-step approach [24–26]. The method starts with all patients and proceeds by repeated splits of patients into two descendent subsets. For this purpose, data from subjects with Decreased and Absent MAD histochemical stainings were grouped together to define an “Abnormal” MAD activity group. The performance of a classifier expressed by its true positive rate (sensitivity, se) and false positive rate (1-specificity, 1-sp) to discriminate between two subclasses of subjects was plotted in a ROC space. Area under the ROC curve (AUC) and the corresponding 95% confidence interval (CI) were calculated to describe the overall performance of classifiers to correctly identify the different MAD activity groups [22,23]. Threshold cut-off values were defined by the points representing the highest concomitant sensitivity and specificity [22,26]. The diagnostic performances of the cut-off and decision tree were evaluated using accuracy, predictive values, likelihood ratios (LRs), and diagnostic odds ratio [24].

## Results

### Subject characteristics

In this observational study, fifty-one patients underwent both the index and standard tests, which were the metabolic exercise testing and muscle biopsy, respectively, with a time interval of 86.2 ± 44.6 days. No adverse event was observed during or following the index test. The patients who performed the index test were referred by ten different clinicians, including neurologists, internal medicine specialists, rheumatologists, and sport medicine specialists. The main symptoms and laboratory findings of subjects are shown in Table 1.

**Table 1:** Clinical features and laboratory findings of patients.

GSD	MAD activity	Patient No./sex/age	Exertional symptoms			Post-exercise Symptoms		Other symptoms	Biology			
			Muscle weakness	Myalgia	Cramps	Myalgia	Cramps		CK	Free carnitine / Total carnitine	Acylcarnitine profile	Urine organic acids profile
**McArdle**	**Normal**	1/F/17	+/ 7 yrs	+/ 7 yrs	-	-	-	Second wind +	5 x ULN	ND	ND	ND
**Tarui**	**Normal**	2/F/37	+/ since childhood	+/ since childhood	+	+/ since childhood	+	Myoglobinuria, second wind –	44 x ULN	ND	ND	ND
**McArdle**	**Decreased**	1/F/47	+/ since childhood	-	-	-	+	Second wind +	3 x ULN	0.6 LLN / 0.7 LLN	N	N
**Absence of GSD**	**Normal**	1/F/35	+/ 6 yrs	+	-	-	-		N	N / N	N	N
	**Normal**	2/M/17	-	+/ 7 yrs	+	+/ 3 yrs	+	CK up to 9000 UI/L	N	N / 0.9 LLN	N	N
	**Normal**	3/F/38	-	+/ 6 mths	-	-	-		N	ND	ND	ND
	**Normal**	4/M/46	+/ 6 mths	+/ 6 mths	-	+/ 6 mths	-		N	N / N	N	N
	**Normal**	5/M/22	-	+/ 2 yrs	-	-	-	2 episodes of rhabdomyolysis (CK up to 56000 UI/L), second wind +	N	0.8 LLN / 0.7 LLN	N	N
	**Normal**	6/M/57	-	+/ 3 yrs	-	-	-		N	ND	ND	ND
	**Normal**	7/M/38	+/ 2 yrs	-	-	-	-		N	N / N	N	N
	**Normal**	8/M/56	-	+/ 2 yrs	-	-	-		N	N / N	N	N
	**Normal**	9/M/17	-	+/ 4 yrs	-	+/ 4 yrs	-		1.5 x ULN	N / N	N	N
	**Normal**	10/M/45	-	+/ 2 yrs	+, main symptom	-	-	-	N	N / N	N	N
	**Normal**	11/F/31	+/ 5 yrs	+/ 3 yrs	-	-	-		N	N / N	N	N
	**Normal**	12/F/46	-	+/ 10 yrs	-	-	-		N	N / N	ND	ND
	**Normal**	13/F/41	-	-	-	+	-	Deafness, suspicion of mitochondrial myopathy	N	N / N	N	N
	**Normal**	14/M/27	+/ since childhood	-	-	-	-		N	N / N	N	N
	**Normal**	15/M/16	-	+/ 1 yr	+	+	-		N	N / 0.8 LLN	N	N
	**Normal**	16/M/38	-	+/ 4 yrs	-	-	-		N	N / N	N	N
	**Normal**	17/M/50	+/ 2 yrs	+/ 2 yrs	-	+	-		1.5 x ULN	N / N	N	N
	**Normal**	18/M/33	+/ 2 yrs	+/ 2 yrs	-	-	-		N	N / N	ND	ND
	**Normal**	19/F/42	-	+/ 10 yrs	+	-	-		N	N / N	N	N
	**Normal**	20/M/51	-	+/ 10 yrs	-	+/ 10 yrs	-		N	N / N	N	N
	**Normal**	21/M/41	+/ since childhood	+	-	-	-		N	N / N	ND	ND
	**Normal**	22/M/41	-	+/ 20 yrs	-	-	-		N	N / N	N	N
	**Normal**	23/M/53	-	+/ 15 yrs	-	+/ 15 yrs	-		3 x ULN	N / N	N	N
	**Normal**	24/F/20	-	+/ 2 yrs	+	-	-	2 episodes of rhabdomyolysis (CK up to 50000 UI/L)	N	0.6 LLN / 0.5 LLN	N	N
	**Normal**	25/F/44	+/ 1 yr	+/ 1 yr	-	+	-		N	0.9 LLN / 0.7 LLN	N	N
	**Normal**	26/M/62	+/ 8 yrs	+/ 8 yrs	-	-	+	Myalgia following rosuvastatin therapy	1.5 x ULN	N / N	N	N
	**Normal**	27/M/28	-	+/ 8 yrs	-	+/ 1 yr	-	1 episode of rhadbdomyolysis (CK = 14000 UI/L) and myoglobinuria	1.5 x ULN	N / N	N	N
	**Normal**	28/M/20	+/ 1 yr	+/ 1 yr	-	-	-	1 episode of rhadbdomyolysis (CK = 40000 UI/L)	N	N / N	N	N
	**Normal**	29/M/18	+/ 1 yr	+/ 1 yr	-	-	-		N	N / N	N	N
	**Normal**	30/M/18	-	-	+	-	-		N	0.4 LLN / 0.3 LLN	N	N
	**Normal**	31/M/51	-	+/ 2 yrs	+	-	-		2 x ULN	ND	ND	ND
	**Normal**	32/M/16	+/ 1 yr	-	-	+/ 1 yr	-		N	0.8 LLN / 0.7 LLN	N	N
	**Normal**	33/M/51	-	+/ since childhood	-	-	-		N	0.9 LLN / 0.8 LLN	N	N
	**Normal**	34/M/20	-	+/ 2 yrs	-	-	-	Episodes of rhabdomyolysis	N	ND	ND	ND
	**Normal**	35/M/16	-	+/ 1 yr	+	+/ 1 yr	-		2 x ULN	N / N	N	N
	**Normal**	36/F/34	+/ 2 yrs	+/ 2 yrs	-	+/ 2 yrs	-	Ptosis, diplopia	N	0.9 LLN / 0.8 LLN	N	N
	**Normal**	37/F/27	-	+/ 4 yrs	-	-	-		N	N / N	N	N
	**Decreased**	1/F/43	+/ 2 yrs	+/ 2 yrs	-	+	-		N	N / N	N	N
	**Decreased**	2/M/33	-	-	+	-	+		3 x ULN	N / N	N	N
	**Decreased**	3/F/50	-	+/ 5 yrs	-	+/ 5 yrs	-		N	N / N	N	N
	**Decreased**	4/F/39	-	+/ 20 yrs	-	+	-		N	N / N	N	N
	**Decreased**	5/F/46	-	+/ 7 yrs	-	+	-	1 episode of rhadbdomyolysis and myoglobinuria	N	0.9 LLN / 0.7 LLN	N	N
	**Decreased**	6/F/28	-	+/ 1 yr	+	+	+		N	0.6 LLN / 0.5 LLN	N	N
	**Absent**	1/F/29	+/ 10 yrs	+	+	+	+		N	0.7 LLN / 0.7 LLN	N	N
	**Absent**	2/M/16	+/ 2 yrs	-	+	-	-		2 x ULN	ND	ND	ND
	**Absent**	3/F/60	+/ 15 yrs	+/ 15 yrs	-	+	-	Myoglobinuria	N	N / N	N	N
	**Absent**	4/M/16	+	+/ 7 yrs	-	-	-		N	N / N	N	N
	**Absent**	5/M/42	+	+/ 6 yrs	-	-	-		N	ND	ND	ND

GSD: Glycogen storage disease, MAD: Myoadenylate deaminase, +/: Present/ Duration in years or months,-: Absent, CK: Creatine kinase determined prior to exercise testing, N: Normal value, LLN: Lower limit of normal, ULN: Upper limit of normal, ND: Not determined.

**Fig 1:**
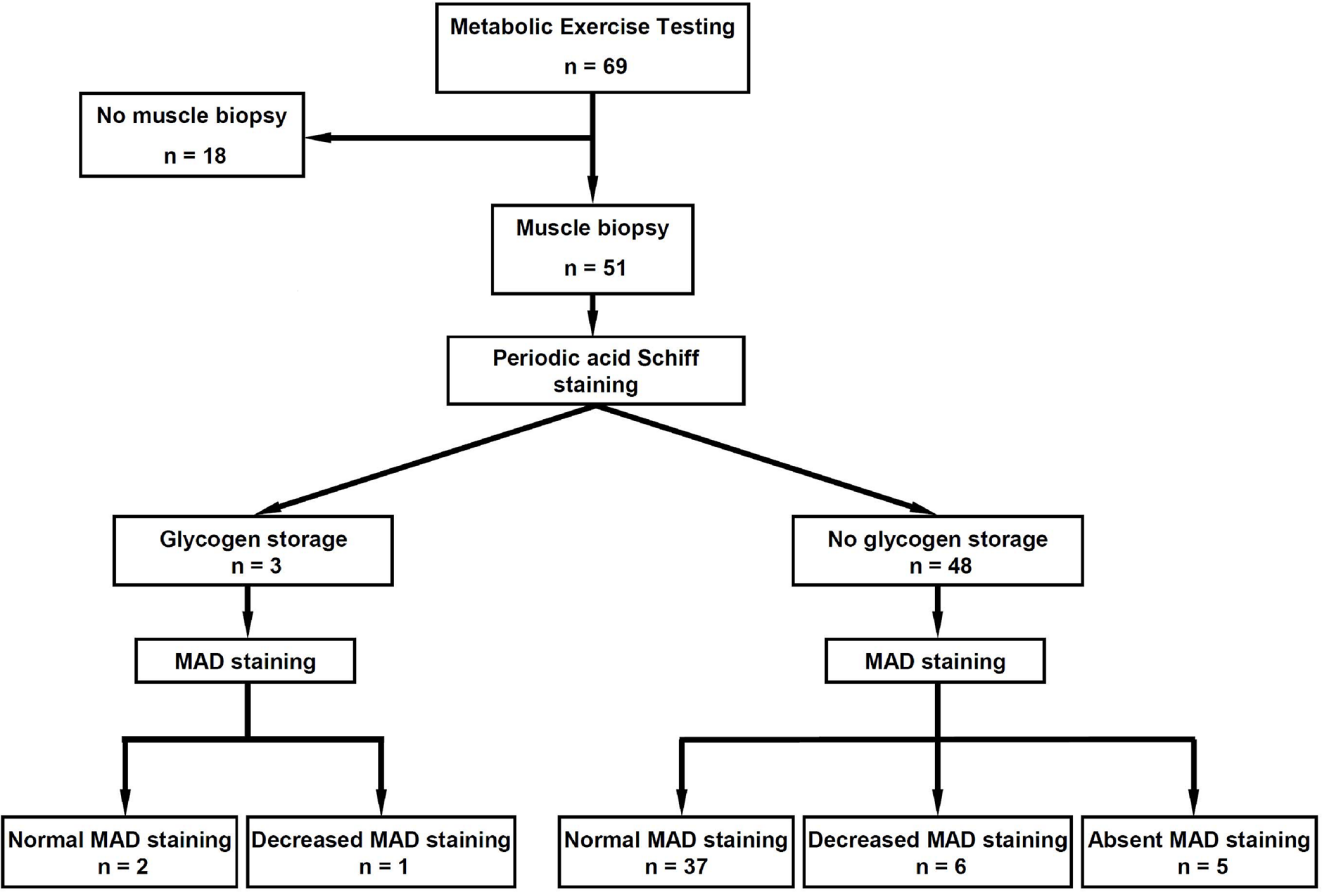
Flowchart of the study. Muscle biopsies were analysed using standard techniques including histoenzymology with Periodic acid-Schiff (PAS) and MAD staining (Fishbein’s method [35]).

In this cohort, the observed metabolic myopathies were glycogenoses and MAD deficiencies. A flowchart summarizing the study is given (Fig 1).

**Fig 2:**
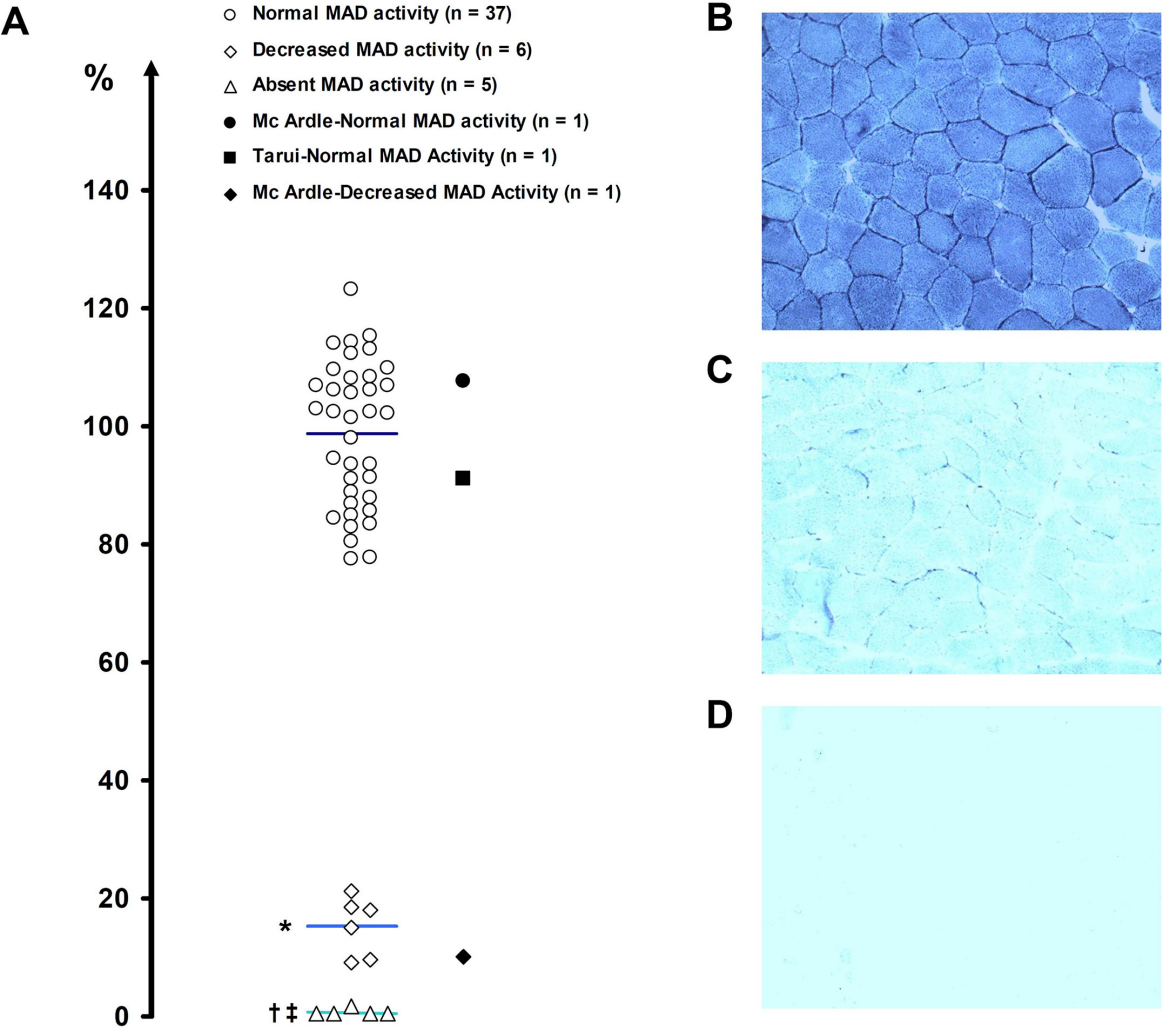
Histochemical MAD staining using *p*-nitro blue tetrazolium. (A) Relative *p*-NBT staining intensity was expressed as a percentage of the mean optical density in control muscle biopsies (see Methods). Horizontal bars represent the mean value of each group. *: Decreased vs. Normal MAD staining, †: Absent vs. Normal MAD staining, ‡: Absent vs. Decreased MAD staining (*P* < 0.05, Games-Howell *post-hoc* test). Representative serial cross sections of lateral vastus biopsies: (B) Normal, (C) Decreased, and (D) Absent MAD staining (original magnification: × 100).

Subsarcolemmal glycogen accumulation at PAS staining was present for three subjects. In this subgroup (glycogenoses), the diagnosis for McArdle (n = 2) and Tarui (n = 1) diseases was confirmed by the absence of myophosphorylase and phosphofructokinase activity, respectively, in biochemical analysis. Features of the subject presenting Tarui disease have been presented elsewhere [41]. Fig 2 displays the results of histochemical staining in muscle sections using the *p*-NBT reaction. One patient had a double enzyme defect involving myophosphorylase and MAD activities. In the 48 patients presenting no glycogen storage, the analysis of scatter plot distribution (Fig 2A) indicates three groups of subjects (*P* < 0.0001, ANOVA) according to Absent (n = 5), Decreased (n = 6) or Normal (n = 37) MAD staining. A specimen set of stained quadriceps biopsies is shown for each group (Fig 2B–2D).

Anthropometric and exercise testing data of included subjects are listed in Table 2.

**Table 2:** Anthropometric characteristics and maximal exercise test data.

	No glycogenoses			Glycogenoses		
				McArdle	Tarui	McArdle
MAD activity	Normal	Decreased	Absent	Normal	Normal	Decreased
**Number (n)**	37	6	5	1	1	1
**Sex (f/m)**	10/27	5/1	2/3	f	f	f
**Age (years)**	35.3 ± 13.9	39.8 ± 8.2	32.6 ± 18.7	17	37	47
**Weight (kg)**	69.6 ± 14.2	61.5 ± 14.0	77.8 ± 20.4	55	65	54
**BMI (kg.m** ^**-2**^ **)**	23.6 ± 3.9	22.4 ± 3.4	27.5 ± 10.0	20.7	28.1	22.2
**% Predicted peak power**	92.8 ± 18.7	99.1 ± 27.8	75.4 ± 23.0	48.0	62.7	73.8
**Peak V’O**_**2**_ **(ml.min** ^**-1**^ **.kg** ^**-1**^ **)**	34.6 ± 9.4	30.8 ± 10.8	30.9 ± 16.3	21.8	14.5	20.2
**% Predicted peak V’O**_**2**_	100.2 ± 15.6	102.3 ± 24.9	93.6 ± 24.3	61.7	52.9	76.1
**Heart Rate at end-exercise (beats/min)**	171.1 ± 16.8	158.5 ± 21.4	144.8 ± 22.6	182	153	173
**% Predicted maximal heart rate**	92.6 ± 6.9	88.2 ± 13.2	77.1 ± 6.3*	89.7	83.6	97.7

Data are means ± SD. MAD: Myoadenylate deaminase, f: Female, m: Male, BMI: Body Mass Index

*: Different from the Normal MAD activity group (*P* < 0.05, Games-Howell *post-hoc* test).

There were no significant differences in the mean age and body mass index (BMI) between the MAD activity subgroups, and no distinction could be made on the basis of maximal O_2_ uptake (expressed as percentage of predicted maximal O_2_ consumption, %PV’O_2_; ANOVA, *P* = 0.691).

### Plasma metabolites

**Fig 3:**
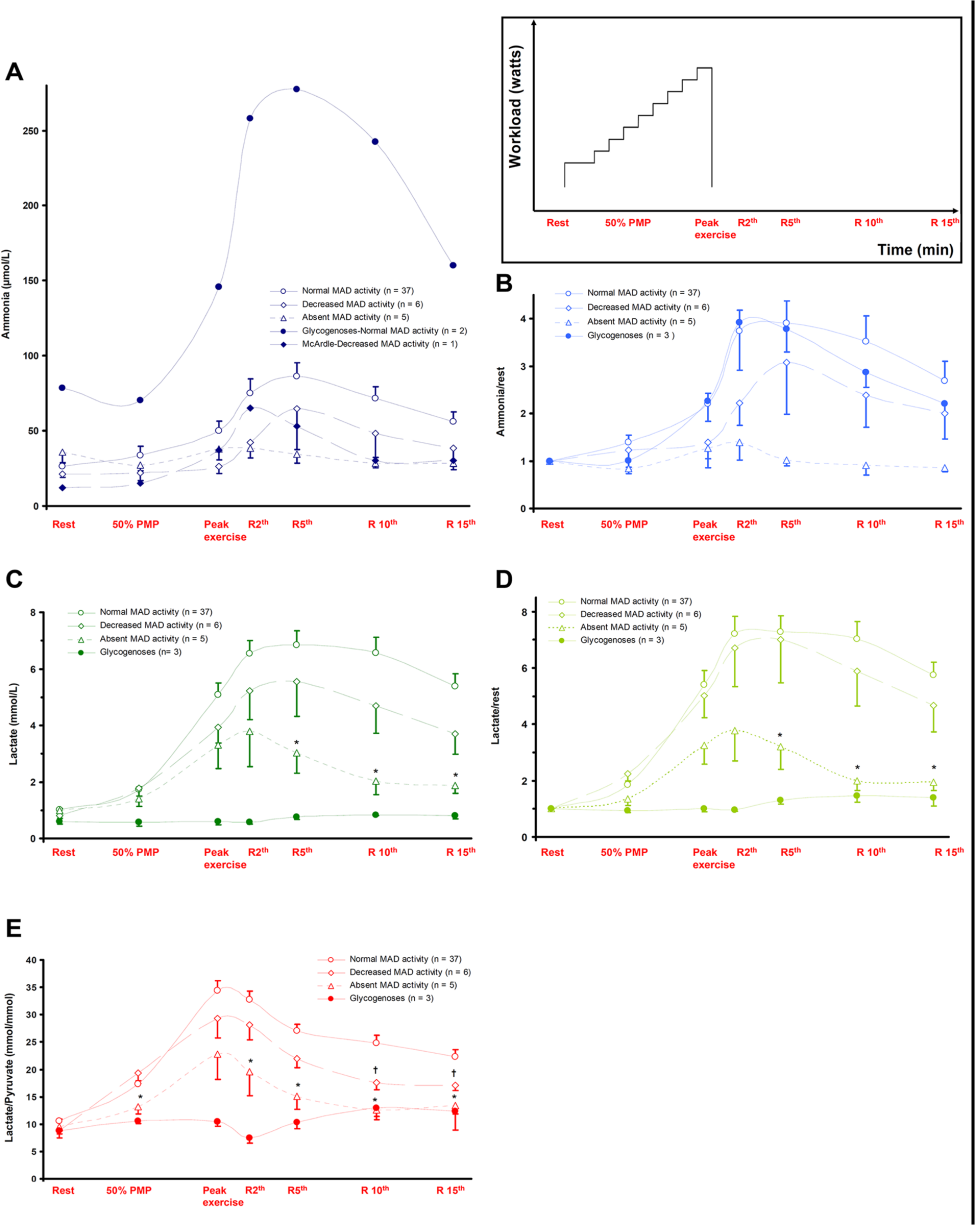
Effects of incremental exercise on plasma metabolite levels according to muscle MAD activity. Patients performed an incremental exercise testing (*inset*). Blood was sampled before (Rest), during exercise (50% of Predicted Maximal Power and Peak exercise), and after exercise (2, 5, 10 and 15 min recovery). Filled symbols correspond to glycogenoses, open symbols correspond to the absence of glycogenoses. (A) Ammonia. (B) Ammonia/rest. (C) Lactate. (D) Lactate/rest. (E) Lactate/Pyruvate ratio. Data are represented as means ± standard error of mean (error bars not included for ammonia values in the subgroup with glycogenose and normal MAD in panel A).*: Absent vs. Normal MAD staining, †: Decreased vs. Normal MAD staining (*P* < 0.05, Games-Howell *post-hoc* test).

Fig 3 shows the effects of exercise on plasma concentration of metabolites. The highest values for ammonia and lactate are reached during recovery from exercise. Because of the small number of subjects in the glycogenoses subgroup, their data were not included in the statistical analysis.

Plasma ammonia concentrations determined before, during, and after exercise are displayed in Fig 3A. As the pre-exercise levels for ammonia were different between the groups and exercise increases its plasma concentration, data were normalized to rest values (Fig 3B). The results of the analysis of variance (*P*) were 0.0848 and 0.0918 at the 10^th^ minute and the 5th minute of recovery, for ammonia and ammonia/rest values, respectively.

Changes in plasma lactate and lactate/rest during and after exercise are shown in Fig 3C and 3D. In the Absent MAD activity group, the rise in plasma lactate concentration was significantly lower than that of the Normal MAD activity group (*P* < 0.05, Games-Howell *post-hoc* test).

Lactate/pyruvate ratio (L/P) changes during and after exercise are shown in Fig 3E. At 50% PMP, L/P was 13.2 ± 2.9 mmol/mmol in Absent and 17.3 ± 3.5 mmol/mmol in Normal MAD activity groups (*P* < 0.05, Games-Howell *post-hoc* test). Throughout recovery, L/P was significantly different between Absent and Normal MAD activity groups (*P* < 0.05). Differences in L/P between Decreased and Normal MAD activity groups (*P* < 0.05) were observed during late recovery from exercise (10 and 15 min).

### Normal Vs. Abnormal MAD activity, and Decreased Vs. Absent MAD activity

**Fig 4:**
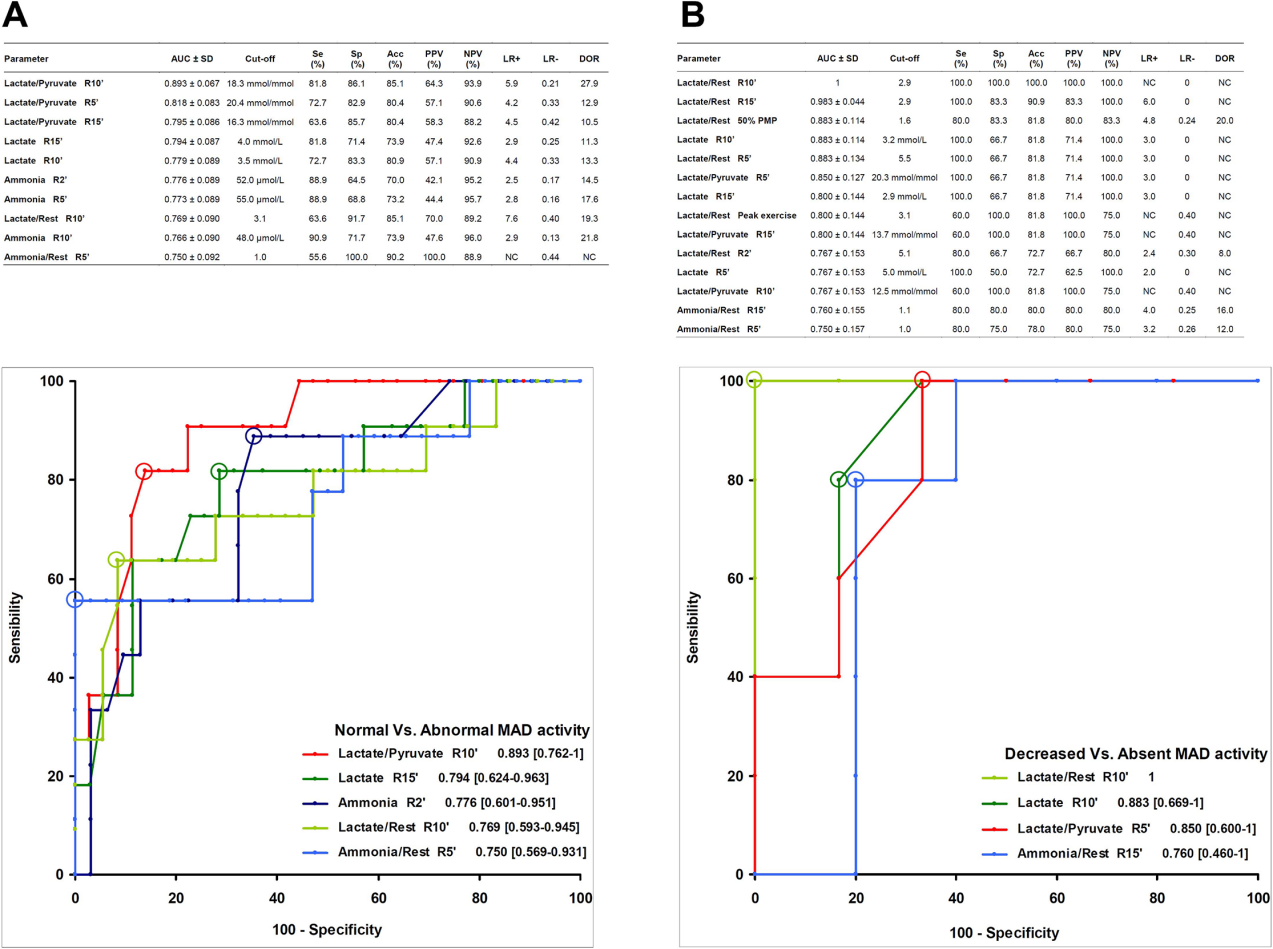
Diagnostic performance of blood parameters to discriminate abnormal from normal MAD activity (A) and decreased from absent MAD activity (B). Diagnostic indices for predictors, with areas under receiver operating characteristic curves (AUC) ≥ 0.750 listed in the tables. The best AUC for each parameter is shown in the ROC space, with the corresponding 95% confidence interval. Circles denote cut-off points, corresponding to the highest concomitant sensitivity and specificity. (A) Performance of blood parameters to discriminate between Normal (n = 37) and Abnormal (n = 11) MAD activity. (B) Classifiers for the differentiation of Absent (n = 5) from Decreased (n = 6) MAD activity. 50% PMP: 50% from predicted maximal power (see Methods), R: Recovery from exercise, Se: Sensibility, Sp: Specificity, Acc: Accuracy, PPV: Positive predictive value, NPV: Negative predictive value, LR+: Positive likelihood ratio, LR-: Negative likelihood ratio, DOR: Diagnostic odds ratio, NC: Not calculable.

Fig 4A summarizes the diagnostic performance of classifiers for the differentiation of Abnormal from Normal MAD activity. L/P at the 10^th^ minute of recovery demonstrates the best diagnostic efficacy to discriminate Abnormal from Normal MAD activity with an AUC value of 0.893 (95% CI 0.762−1). The optimal cut-off is 18.3 mmol/mmol, with a sensitivity of 81.8% and a specificity of 86.1% for detecting Abnormal MAD activity.

In the next step, we sought to distinguish Absent from Decreased MAD activity (Fig 4B). Lactate/rest at the 10^th^ minute of recovery gives an optimal AUC (1) with a discriminator value of 2.9.

### Algorithm

**Fig 5:**
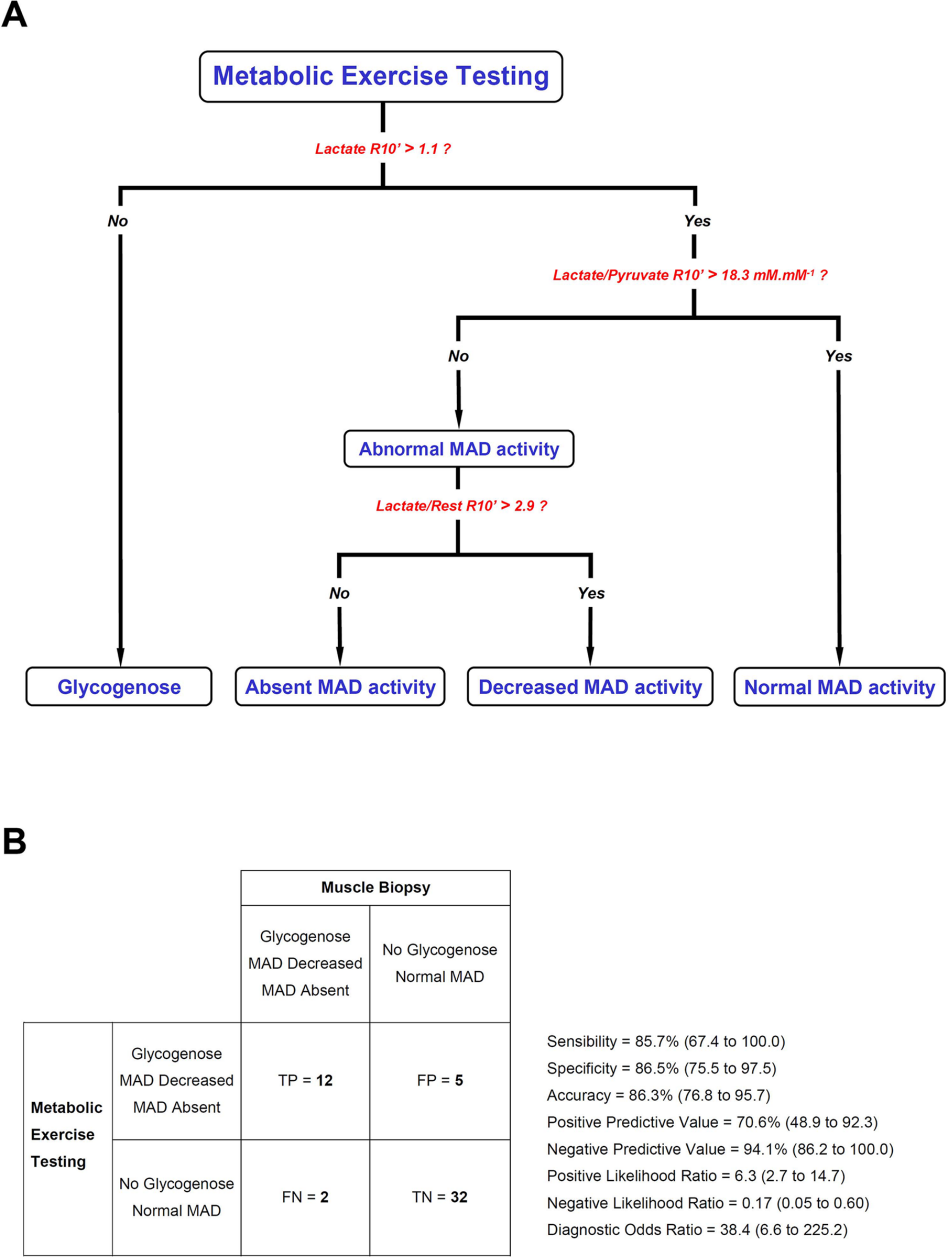
Diagnostic algorithm for glycogenoses and MAD deficiencies. (A) The algorithm classifies subjects referred for metabolic exercise testing into four groups. The figure combines the optimal cut-off values reported in decision tree nodes (Fig 4A and 4B). L/P: lactate-to-pyruvate ratio, R: Recovery from exercise. (B) A contingency table was constructed on the basis of whether subjects have a metabolic myopathy or not, and corresponding common performance metrics with 95% confidence interval were calculated. TP: True positive, FP: False positive, FN: False negative, TN: True negative.

Glycogenoses were introduced into the model, using the lactate concentration at the 10^th^ minute of recovery as mean+2SD to set cut-off value. By adding the two above decision tree nodes (Fig 4A and 4B), the resulting diagnostic algorithm for metabolic myopathies (Fig 5A) yields a diagnostic odds ratio of 38.4 and an accuracy of 86.3% (Fig 5B).

## Discussion

Muscle diseases often pose diagnostic challenges, even to expert clinicians. Given the considerable overlap in the clinical manifestations of patients with metabolic myopathies and those with other muscle disorders, this study emphasizes the rationale to examine patients through exercise testing. In this research field (i.e. metabolic myopathies and bicycle exercise test), blood sampling from an antecubital vein while the legs are exercising is widely used [6,7,9,31,32]. From a physiological point of view, blood samples from femoral vein would provide a better picture of muscle metabolism during exercise on cycloergometer. However, it should be emphasized that blood sampling from the femoral vein during bicycle exercise is more challenging from a practical point of view. Despite these methodological considerations, blood samples from antecubital vein performed between the 5^th^ and the 10^th^ minute following maximal exercise can generate a wide range of metabolites concentration values, thereby enabling the identification of enzyme defects. The subsequent histochemical analysis of muscle biopsy should therefore focus on the respective enzymes for reducing muscle sample size and avoiding unnecessary analysis [1,4].

To determine the sensitivity and specificity of plasma biomarkers sampled during CPX for MADD diagnosis, *p*-NBT staining of muscle sections was defined as the reference test. The broad spectrum of MAD staining intensity, from absent to heavy blue stippling, illustrates the high specificity of Fishbein’s method and supports its routine utilization in pathology laboratories [16,35]. MAD deficiency was the only observed abnormality in the Absent and Decreased subgroups, since additional neuromuscular diseases were ruled out by combined clinical, electromyographic, laboratory, and histological examinations. Thus, our cohort of subjects presenting absent and decreased *p*-NBT staining corresponds to primary MAD deficiency according to the classification proposed by Fishbein [12,37]. Currently, the clinical relevance of MAD deficiency is still a matter of debate [37,40,42]. Previous series of patients were based on muscle biopsy collection. We hypothesized that another muscle disease to which the biopsy relates may easily mask as well as initiate a MADD [16,17,39,43] for several reasons. First, it should be emphasized that the maximal aerobic performance, i.e. maximal oxygen uptake and power, is preserved in this enzyme defect. Additionally, recent evidence indicates that MAD activity can be significantly decreased in non-metabolic disorders, even in the absence of muscle inflammation [44,45]. From a physiological perspective, one of the most striking results of this study is that glycolysis parameters, i.e. lactate and L/P, provide the most informative classifiers to discriminate among MAD activity groups. This means that, although MADD leads to decreased ammonia production during exercise, glycolysis is also modulated in this enzyme defect. Lower levels for lactate and pyruvate following maximal exercise testing [9,13] and during forearm ischemic test [5,19] have been previously reported in MADD. Conversely, high ammonia values in glycogen storage myopathies as observed here and previously [5,46], indicate a functional link between the purine nucleotide cycle and the glycolysis pathway. While it has been shown that ammonia stimulates PFK [47], the lower increase in lactate/pyuvate ratio in MADD also suggests a modulation in lactate dehydrogenase activity or a lower [NADH,H^+^]/[NAD^+^] ratio [10].

Whilst an overall 1−3% frequency in MADD has been reported in biopsy series from pathology laboratories [16,39], we found a higher proportion in this study (9.8 and 13.7% for absence and decreased MAD staining, respectively). It should be underlined that physicians refer patients for exercise testing when a metabolic myopathy is suspected, that is when subjects experience exertional myalgia. This may lead to increase the prevalence of metabolic myopathies in a cohort from a department of clinical physiology that carries out exercise tests [16]. At each node of the decision tree, classifiers provide AUC values at least equal to 0.893. As previously proposed [21,22], this corresponds to “highly accurate” discriminators, which strengthens the current algorithm. A further advantage of the present algorithm relates to its property of encompassing glycogenoses and MAD defects using only two blood samples, i.e. rest and the 10^th^ minute of recovery. The diagnostic parameters of a test are critically dependent upon the clinical context within which they are employed. For example, a clinician can refer patients to confirm the inability of muscle to produce lactate and subsequently search for the common genetic mutation for McAdle disease. Conversely, some clinicians can use the exercise test to eliminate a metabolic myopathy in subjects with exercise-induced myalgia. In this regard, LRs are more intuitive for altering disease probability compared to sensitivity and specificity. Using the present LRs (Fig 5B) and Fagan’s nomogram, the post-exercise test probability of having or not a metabolic myopathy can be easily calculated without performing the reference test (i.e. muscle biopsy).

**Fig 6:**
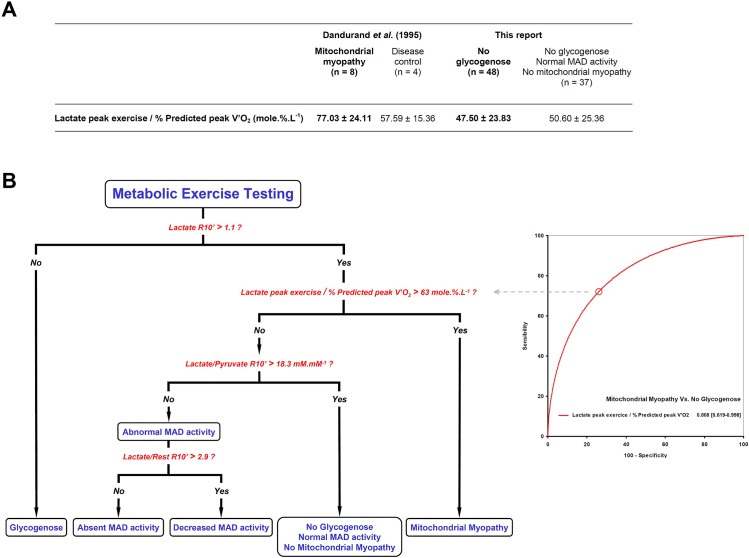
Proposed algorithm for the diagnosis of glycogenoses, MAD deficiencies, and mitochondrial myopathies. The ROC curve to discriminate between the presence and absence of mitochondrial myopathy was determined according to the parametric methodology [22]. (A) For this purpose, we used the values (mean ± SD) published by Dandurand *et al*. in eight patients with mitochondrial myopathies [51]. The values in the disease control group from [51] are in line with those from the group with no metabolic myopathy (n = 37) in the present study. (B) The cut-off corresponds to the highest value for the Youden index (Se = 72.5%, Sp = 73.5%). MAD: Myoadenylate deaminase.

According to the methodological standards for diagnostic studies, all the consecutive referred patients were included. In spite of this, we found no mitochondrial myopathy in our cohort. It should be emphasized that symptoms involving different organs or systems are the hallmark of mitochondrial respiratory chain deficiencies [32,48,49,50]. To date, incremental exercise testing has been evaluated in selected subjects with well-characterized mitochondrial myopathies [7,32,49,51,52], in contrast with our prospective methodology. The apparent discrepancy between our results and the existing literature may be related to differences in the recruitment methodology. Except for two patients with deafness and diplopia, the presenting symptoms and clinical outcomes in our cohort were restricted to skeletal muscle or exercise-induced, thereby reducing the probability of mitochondrial myopathies. Therefore, we consider that the present algorithm (Fig 5A) is more appropriate for managing patients with isolated skeletal muscle symptoms. During exercise, mitochondrial myopathies are featured by an impairment in the oxidative phosphorylation of skeletal muscle, higher plasma lactate concentrations, or, most often, a combination of both [7,8,11,49,51,52,53,54]. Accordingly, we propose a supplemental algorithm which includes mitochondrial myopathies using the lactate value at peak exercise normalized to the percentage of predicted maximal O_2_ uptake (Fig 6). The cut-off was calculated according to the parametric approach for ROC curves [22] by using the values for the mean and the standard deviation in patients suffering from mitochondrial myopathies from the study of Dandurand *et al*. [51]. Even though further studies with subjects suffering from mitochondrial myopathies are needed to refine the value of this cut-off, our proposed parameter (lactate peak exercise / % predicted peak V’O_2_) is both physiologically relevant and consistent with previous reports [7,11,32,49,51,52,53,54].

## Conclusion

In summary, the current algorithm provides a non-invasive diagnosis for MAD deficiency, offering the prospect of a valuable aid both for selecting patients for muscle biopsy and selecting the appropriate histochemical analysis methods.

## Supporting Information

S1 File STARD Checklist. (DOC)

S2 File Data set. (XLS)
